# Supplementary material for: Causes and risk factors for an acute kidney injury outbreak among children in The Gambia, June – September 2022: A case-cohort study
Source: PLoS One. 2025 Jun 12;20(6):e0324931. doi: 10.1371/journal.pone.0324931 (PMC12161566; doi:10.1371/journal.pone.0324931)
Supplement: Attachment — Submitted filename: RESPONSE_REVIEWERS_AKI_PLOS ONE_23.08.24.docx [file pone.0324931.s005.docx]

**Table 2x2: Response to the reviewer’s comments**

| **Comments** | **Responses** | **Page** |
| --- | --- | --- |
|  |  |  |
| **Additional Editor Comments:** |  |  |
| Thank you for submission of this manuscript: Causes and risk factors for an acute kidney injury outbreak among children in The Gambia, June – September 2022: A case-cohort study (PONE-D-24-22500) Although the topic is interesting, there are several issues that must be corrected before further processing of this manuscript can be done. | | |
| Abstract 1. No study objective written | Thank you for pointing that out. We have provided an objective in both the abstract and at the end of the introduction | Page 3, lines 44-45; Page 5, lines 96-98 |
| Introduction 1. Add more data and update about death toll number, fatality rate, what kind of medications, what substances cause this issue (DEG/EG) (all available here: <https://www.kidney-international.org/article/S0085-2538(22)01025-0/fulltext>) | We have confirmed from the latest linelist as of 03^rd^ of December 2022 that the number of confirmed deaths was 66. We can provide the linelist on request. As investigators on the ground, we attended Incident Management Team meetings headed by the MoH of the Gambia where the number of deaths was confirmed to be 66.  We have added information on the medications that was suspected to cause that issue. | No changes made  Page 4, lines 74, 75 |
| 2. No citation from line 69-79, please add more | Thank you for your comment. We have provided more citations | Page 4, lines 71-81 |
| 3. Reference 5 is missing | We have added the missing reference which is now reference number 9 | Page 4, line 71 |
| Methods 1. No need “” in STROBE | We have removed the “” | Page 5, lines 101, 102 |
| 2. Add primary data collection in addition to “the national epidemiological surveillance system, line-list and preliminary epidemiological reports” | Thank you for your comments: We have reviewed as such: “We collected primary data and reviewed data from the national epidemiological surveillance system, line-list and preliminary epidemiological reports” | Page 5, line 109; Page 6, lines 110-11 |
| 3. Update DHS data to 2019-2020 (<https://dhsprogram.com/publications/publication-FR369-DHS-Final-Reports.cfm>) and write the interpretation | We have updated the data source and provided the reference | Page 7, lines 120, 121 |
| 4. Add definition of suspected, probable, and confirmed AKI cases in addition to general AKI definition | We have added definitions as suggested | Page 6, lines 132-139 |
| 5. Add proper citation for SAS as analytical software | We have added a proper citation for the SAS software | Page 7, line 146 |
| 6. Any explanation about controls who may become cases (no AKI is the inclusion of control) | Thank you for your comment. In this study no control became a case. We accounted for it in the sample size calculation in case it happened, but it did not happen. We have specified that under the Participants section in the results | Page 7, line 147 |
| 7. Please rewrite: All 82 confirmed cases were sought for inclusion in the study (contradictive since the authors stated that they cannot include every case due to financial constraints) | Thank you for your comment. We have reformulated the sentence and emphasized that the final constraints referred to the inclusion of suspected/probable cases from other regions but did not refer to confirmed cases | Page 7, line 138-140 |
| 8. Eleven patients from suspected and probable cases? Please explain from many cases of this group | Thank you so very much for the comment. We have revised the sentence to clarify. WR1 and WR2 produced a total of eleven suspected/probable cases who were all included in the study. Hence, no sampling was done. | Page 8, lines 152-155. |
| 9. It is vague: probability sampling technique (there are many specific techniques, such as cluster sampling), please correct it | We have specified the sampling technique used. Controls we selected using a simple random sampling technique | Page 7, line 155 |
| 10. Add the Gambia income determination (quantitative threshold such as household annual income and income per capita is more suitable, not only about pipe – add reference only if there is no reference to the quantitative determination of income) | Thank you for the very important comment. We provided details on the determination of the income and the methodology we used. | Page 9, lines 185-196 |
| 11. Looks like there is more than 3:1 control:cases (nearer to 4:1) | We enrolled all the controls planned for but some of the cases declined to participate in the study. Hence, the distortion in the ratio of cases to controls.  This does not affect the power of our study. |  |
| Result 1. It is inconsistent, where is the 82 confirmed cases as mentioned earlier? | Thank you for your comment. We recruited 82 confirmed cases. Of these, we enrolled 58 and analysed 57 together with 6 suspected and probable cases to make a total of 63 cases. | Page 11, Flow chart |
| 2. Quranic education is not a form of formal education, consider to merge with no education | For the inferential analysis, the education variable was categorised into binary; High and Low. Primary, Quranic and None formed the low category. According to the literature, health literacy is congruent with secondary and higher education. |  |
| 3. What is the threshold for younger child? Two Gambian children with a one-year difference seems not standard | Thank you for your comment. There is no threshold for younger child, The variable “Age” was considered as continuous (in year) in the analyses. The models compared two individuals with X and (X+1) as ages respectively. The younger child here will be the one with X year old. |  |
| Discussion 1. Add citation in the first paragraph | We added some citations as suggested | Page 16, line 260, 265 |
| 2. Is there any specific measure that already done by the government (in addition to withdrawing the drug) – to prevent similar event in the future | Thank you for the comment. The products of the Company (Maiden pharmaceuticals Limited) were banned to be imported in the Gambia and the company closed. Internally, the government increased the control at the port of entry in the Gambia. Included in the introduction. | Page 4, lines 86-88 |
| Figure  1. From where is figure 1? If from other sources, state the permission to publish, while if created by the authors, please add about software utilized to create it | We have provided the source of the figure 1 and the software used | Page 6, line 117 |
| Table 1&2 1. Do statistical analysis to compare characteristics between each group. Descriptive statistics are not enough – update in the methods | Since we were describing groups, we did not provide p-values as per guidelines such as the STROBE and the CONSORT. It is recommended to use literature review and unbalanced variables identified |  |
| Table 1 1. The authors wrote that they include all 82 patients; however, the one mentioned in the table is only 63 – please explain about this difference | Refer to our response to the comment 1 under Result:  Thank you for your comment. We recruited 82 confirmed cases. Of these, we enrolled 58 and analysed 57 together with 6 suspected and probable cases to make a total of 63 cases | Page 11, Flow chart |
| 2. Is all data have abnormal data distribution? Because ratio is mentioned in median (IQR), please check | Yes, we checked, and the distribution of the continuous variables was not normal. So, we decided to present the median (IQR). | Pages 11,12, table 1 |
| 3. Add MUAC long form | We provided the long form of MUAC as mid-upper arm circumference | Pages 12, table 1 |
| 4. Why is there many missing weight and height (>60?) | Most of the missing data are from the dead cases that we did have the opportunity to measure. We used records to collect these variables which were missing in many of the records |  |
| 5. Remove Relation caregiver – child (mother), n (%) | We have renamed it as it refers to mother as caregiver | Page 12, table 1 |
| 6. Remove Education mother (High school), n (%) 321 90 (34.9) 19 (30.2) | We have removed it from the table | Page 12, table 1 |
| 7. Consider removing: Education head of family, n (%) | We have removed it from the table | Page 12, table 1 |
| Funding |  |  |
| 1. Add funding number | We have provided funding number as suggested | Page 20, line 361 |
| **5. Review Comments to the Author**  Please use the space provided to explain your answers to the questions above. You may also include additional comments for the author, including concerns about dual publication, research ethics, or publication ethics. (Please upload your review as an attachment if it exceeds 20,000 characters)  Reviewer #1: Incidences like the one which happened in Gambia expose the perils of adulterated medicine and begs better regulatory oversight and control measures to prevent such incidences. Authors have done an epidemiological investigation to confirm the cause and contributory risk factors. I have following comments: | | |
| Material and methods need more clarity.  Authors state that cases and controls were selected from the main cohort. Were all the cases of AKI in the region during the study period selected? Or was it studied in only the main cohort which as per the study is only 625 patients. How was the main cohort selected? | Thank you for your comment. Yes, all the cases in the regions were selected.  The main cohort comprised all the children that resided in the villages from which the cases arose.  The sub-cohort was as a fraction of the main cohort determined by proportionality to the number of confirmed cases in a given village. | Page 5, lines 11-102 |
| How did the authors ensure that all the cases of AKI were selected? | We worked from a line-list identified and confirmed by the Ministry of Health. | No change |
| What was the study period? It needs to be mentioned. | From 15 to 22 December 2022. | Page 5, line 107 |
| What was the criteria for selecting sub cohort? Why was there a necessity to create a sub cohort? | There were no criteria to select the sub-cohort. The sub-cohort was a fraction of the main cohort that was enough to provide the number of controls needed.  The advantage of the sub-cohort was to ensure cost-effectiveness of the study while ensuring its scientific validity. Using the main cohort would have been very expensive with no extra advantage. |  |
| The flowchart in figure 2 mentions that there were 11 patients who were unreachable and eliminated. Once again it mentions there were 5 more unreachable and number of suspected/probable cases have thus reduced to 6.  This is confusing. Number of cases who were removed because of unreachable should be clubbed together only once. | Thank you for your comment. Lumping them together does not give an accurate picture of the points at which the cases were not reachable. Hence, the decision to separate them in the flow chart. |  |
| AKI definition is not clear. There are clear cut definitions of AKI as per KDIGO. That should be mentioned. | We have added definitions as suggested | Page 6, lines 122-131 |
| Was any biochemical tests done? Like serum creatinine? If yes, then please provide details | Yes, biochemical tests were done namely serum creatinine and urea. But it was serum creatinine that was used in the case definition. An AKI case was diagnosed if the measured creatinine was 1.5-2 times greater than the reference | Page 7, line 133 |
| Please provide detailed table with logistic regression. What factors were adjusted for? There seems to be many confounders. | Thank you so very much for your comment. The logistic regression was used to identify risk factors of AKI. In the identification of risk factors, by design, we do not look for the effect of an exposure on an outcome. Hence, there is nothing like adjusting for confounders. |  |
| The tables need to be redone completely. Under AKI heading sum of so many columns is coming as 64 but the number mentioned is 63. | Thank you for your comment. It is correct. We have redone the table as suggested and provided actual number and percentage. | Pages 11-12, table 1 |
| In Table 2 under the clinical characteristics the N value is varying everytime (e.g. 319, 318,312…) rather than being constant at the number 321. The numbers are not adding up | Thank you so very much for the comment. The table 2 was corrected as well. We have harmonised the totals | Pages 13-14, Table 2 |
| More number of patients who developed AKI had fever, vomiting. Was this before (as a part of disease process) or after giving the medicines? | Yes, these symptoms were developed after exposure to the medicines |  |
| **Reviewer #2**: This is a well written and relevant article. I would suggest a few modifications to the draft to make it useful to readers of the journal Paragraph 1, line 3: Can include obstructive uropathy as other causes of AKI. | We have added as suggested | Page 4, line 65 |
| Line 4 - elaborate why burden of AKI is high in children in sub-saharan Africa | We have elaborated as requested | Page 4, lines 70-71 |
| For secondary exposures - elaborate on concomitant drugs (antibiotics, antimalarial drugs), toxins from animals and industrial toxins - which groups or products were analysed?  Are they associated with AKI? Although the statistical analyses paragraph mentions natural and industrial toxins - no mention of it in results or analyses shared | According to the literature, these are potential causes of AKI. So, we included them in the study assessed by self-report. We did not analyse any product as secondary exposures.  All these variables were included in the treatment model that was used to produce the IPTW for the MSM. The MSM does not assess for associations. The focus for the MSM was the main exposure.  However, using a multivariable logistic regression to identify risk factors, all these variables were entered into the models, but were dropped leaving concomitant drugs that we have shown in the results. | Page 16, table 3 |
| . |  |  |
| Did the authors attempt to examine the time between consumption of adulterated medicines and development of AKI? A similar interval between ingestion and onset of AKI in cases would be an important aspect to prove causation in all cases. | Thank you for your comment. Yes, we have examined the temporary relationship between the exposure and the outcome and ensured the anteriority. We did not capture the exact number of days between consumption and development of symptoms. However, this was captured in the causality assessment at the same time as our study. | Page 17, lines 263-265 |
| A brief description on the pathophysiology of AKI due to DEG in the discussion section would further improve the article. | We have added a brief description as suggested | Page 16, lines 268-272 |
| **Reviewer #3**: The manuscript entitled “Causes and risk factors for an acute kidney injury outbreak among children in The Gambia, June – September 2022: A case-cohort study” discusses the consumption of diethylene glycol and ethylene glycol adulterated medicines manufactured by one pharmaceutical company, leading to acute kidney injury (AKI) in children eight years old or younger. Health providers and the public need to be sensitized about the dangers of adulterated medicines, and the government needs access to resources and expertise in pharmaceutical quality control. Additionally, the definition of AKI should be corrected. It is not accurately defined as the serum creatinine level above normal. Instead, it would be best to use the pediatric AKI definitions such as p-RIFLE, AKIN, or KDIGO. | Regarding the recommendations “Health providers and the public need to be sensitized about the dangers of adulterated medicines, and the government needs access to resources and expertise in pharmaceutical quality control” was already provided under recommendations section.  We have elaborated on the case definitions. | Page 20, lines 359 - 362  Page 7, lines 132-140 |
| **Reviewer #4**: Overall: Bittaye et al report on a case-control study conducted in the Gambia in order to investigate an outbreak of AKI among young children in 2022. They found higher odds of developing AKI among children who had ingested adulterated medicines compared to children who did not.  The study addresses an important event. Concerns remain on how the authors dealt with recall bias especially that data was collected/ interviews were conducted months after the cases and that in the interim there had been countrywide sensitization of communities on the symptoms of illness and the suspected culprit medications? Perhaps a study limitation section could address this concern. | | |
| The authors also need to define better what a confirmed case is and what tests/criteria was used to conclude that AKI was of unknown etiology. Suggest the use of phrases “epidemiological” definition of AKI and “clinical” definition of AKI. | We have elaborated on the case definitions used for the study | Page 7, lines 132-140 |
| Introduction: 1. Line 66-67 “The primary causes of AKI include extrarenal pathology such as prerenal azotemia, specific kidney diseases such as acute interstitial nephritis or non-specific conditions such as nephrotoxicity” - Generally, the term “primary cause” of AKI would imply conditions originating in the kidney. please us alternative phrase - Post-renal causes also important cause in children | We have rephrased the sentence | Page 4, lines 67-71 |
| 2. Line 67-68 “Drug induced nephrotoxicity is the third commonest cause of AKI.3” Not in children. More common causes are infections such as sepsis, malaria, acute gastroenteritis, insect and snake bites, nephrotoxins such as haeme, congenital anomalies of the kidneys etc Kindly review papers below:  -Olowu WA, Niang A, Osafo C, et al. Outcomes of acute kidney injury in children and adults in 347 sub-Saharan Africa: a systematic review. The Lancet Global Health. 2016;4(4):e242-e250. -Macedo E, Cerdá J, Hingorani S, Hou J, Bagga A, Burdmann EA, et al. Recognition and management of acute kidney injury in children: the ISN 0by25 Global Snapshot study. PLoS One. 2018; 13(5):e0196586. pmid:29715307 -Lameire N, Van Biesen W, Vanholder R. Epidemiology of acute kidney injury in children worldwide, including developing countries. Pediatr Nephrol. 2017; 32(8):1301–14. pmid:27307245 -Susantitaphong P, Cruz DN, Cerda J, Abulfaraj M, Alqahtani F, Koulouridis I, et al. World incidence of AKI: a meta-analysis. Clin J Am Soc Nephrol. 2013; 8(9):1482–93. pmid:23744003 | We have rearranged it | Page 4. Lines 68-71 |
| 3. line 69-71: “a pediatric nephrologist at Edward 70 Francis Small Teaching Hospital (ETSTH) – the only teaching hospital in The Gambia – observed an unusual increase in the number of cases of AKI among children from five months to seven years of age.” - A little more detail on the usual numbers of AKI seen at the teaching hospital and how many were observed which caused the pediatric nephrologist to raise alarm? | We agree that this information is important. However, we did not get that data from the Ministry of Health. |  |
| 4. Line 76-78: “Nine samples of medicines taken by children with AKI and sent for toxicological tests were found to contain unacceptable levels of diethylene glycol and ethylene glycol” Were these nine samples the actual ones retrieved from the AKI children or nine samples similar to ones (i.e., same brand, manufacturer) taken by the AKI children? Please clarify | The actual ones retrieved from the AKI children. The sentence has been clarified. | Page 4, line 83 |
| 5. line 74-75 “No toxic substances were detected in other pediatric medicinal products supplied 75 in The Gambia by other pharmaceutical companies.” This sentence should come after line 78 | We have moved the sentence | Page 4, lines 83-85 |
| 6. Line 79-80: “Initial epidemiological investigations conducted by the MoH and US CDC were inconclusive as to the cause7” - Some detail on why this report was inconclusive please? - How does the current study address these reasons e.g the recall bias that was pointed out, the methods used in current study? | The reason why this report was inconclusive has been provided.  Our study used more robust scientific methods of causal inference that enabled us to identify the cause which the other study could not identify | Page 4, lines 84-86 |
| Methods: 1. Line 114-115: “or confirmed with serum creatinine above normal and of unknown etiology, diagnosed within the period June to September 2022.”  Please clarify the criteria for a “confirmed case”. i.e the level of increase above normal that would be considered as AKI. | We have elaborated on the case definitions used for the study | Page 7, lines 132-140 |
| “unknown etiology” – what tests were conducted on the cases to rule out the other AKI etiologies” ? I think a checklist of what was used to rule out usual causes would help. | In our study, we used cases that had been diagnosed by specialists already. |  |
| 2. Line 27 under sampling procedure:” All 82 confirmed cases were sought for inclusion in the study. We also sought to include eleven suspected or probable cases from WR1 and WR2.” How were the cases identified? Examining hospital records? How many hospitals? Or was a national registry used. | Cases on the line – list were identified from records of all hospitals in the country. | Page 8, line 152 |
| 3. Line 142 “The research team comprised surveillance officers that had interacted with the children previously.” When previously? During the MOH/cdc study or when the outbreak was first identified? Would this have been a risk for recall bias? | When the outbreak was first identified. We added a sentence.  We agree that this was a potential risk for recall bias but this was mitigated by training the officers in good research methods before the study commenced and highlighting the danger of this bias. | Page 8, line 167  Page 18, lines 308 - 315 |
| Results: 1. Table 1 and 2, on which narration from line 187-201 is based, do not have p values to help show if the differences in the variables between the cases and controls were statistically significant | Since we were describing groups, we did not provide p-values as per guidelines such as the STROBE and the CONSORT. It is recommended to use literature review and unbalanced variables identified |  |
| 2. How many of the children were dialyzed? | None |  |
| 3. Length of time between onset of anuria/oliguria and access to health facility | Not captured in our study |  |
| Discussion: 1. Line 228-229: “The temporal relationship between the importation of the suspected medicines into 229 the country, their consumption and development of AKI among the children is supportive of this hypothesis.” This is being mentioned for the first time here. Needs to have a citation please or perhaps this should be expanded upon in the introduction section before being mentioned here. | We have expanded upon this in the introduction section. | Page 4, lines 72-75 |
| 2. Did dialysis access have any impact on the high mortality among the cases? | Yes, it did. There was no dialysis machine in the country. Hence, the high mortality |  |
