# Supplementary material for: Causes and risk factors for an acute kidney injury outbreak among children in The Gambia, June – September 2022: A case-cohort study
Source: PLoS One. 2025 Jun 12;20(6):e0324931. doi: 10.1371/journal.pone.0324931 (PMC12161566; doi:10.1371/journal.pone.0324931)
Supplement: Attachment — Submitted filename: comments.docx [file pone.0324931.s003.docx]

Thanks for the rebuttal. I have following comments:

Also, authors should provide the p-value for baseline sociodemographic characteristics to show whether any of the baseline characteristics were different between the two groups.

Please mention what was the criteria for selecting factors for logistic regression analysis.

“In the identification of risk factors, by design, we do not look for the effect of an exposure on an outcome. Hence, there is nothing like adjusting for confounders.” The rebuttal is not right. The cause and effect relationship will be meaningful only if one adjusts for the baseline confounders which are very well present in the study. The groups developing AKI also had higher use of anti-inflammatory drugs, antibiotics, acetaminophen. How do we know that these were not the causes of the AKI rather than the suspected adulterated cough syrups. To say that the adulterated drug was the culprit authors should adjust for these factors in the study. Authors are not looking for just risk factors. They are putting a causal association with adulterated drug and hence it becomes important. “The acute kidney injury outbreak that occurred among children in The Gambia in the period June through September 2022 was **causally** associated with the ingestion of adulterated medicines.”
