# Supplementary material for: Causes and risk factors for an acute kidney injury outbreak among children in The Gambia, June – September 2022: A case-cohort study
Source: PLoS One. 2025 Jun 12;20(6):e0324931. doi: 10.1371/journal.pone.0324931 (PMC12161566; doi:10.1371/journal.pone.0324931)
Supplement: Attachment — Submitted filename: feedback plos one paper 2.docx [file pone.0324931.s004.docx]

Comments on revised article titled “**Causes and risk factors for an acute kidney injury outbreak among children in The Gambia, June – 2 September 2022: A case-cohort study****”**

The manuscript reads very well.

Methods:

1. For the definition of controls: Were these all well children from the community from which the AKI cases originated or were some of them unwell with some other sickness at the time of the outbreak? Kindly clarify this in the definition of controls.
2. Line 129-130: “fever, vomiting, 130 diarrhea, cough, with history of syrup consumption or a child 8 years old”

This was used as one of the three definitions of a suspected AKI case.

This is too imprecise a definition for AKI since no renal specific symptoms are included in this definition. The mere fact that a child consumed syrup would be too imprecise to label them as suspected cases.

How many subjects were included because of this particular definition?

If subjects recruited on the basis of this definition did not have AKI, their inclusion could skew the results.

[ Additionally from table 1/2 some of the controls also had vomiting, diarrhoea, cough and had ingested adulterated medications suggesting that the presence of renal specific symptoms in the definition is important]

Discussion:

1. Line 272-274 “The alternative explanation of E. coli infection is unlikely because only 8% of cases presented with bloody 273 diarrhea which increases the risk of developing serious complications such as acute renal failure. Only two 274 samples tested positive for Shiga toxin-producing E. coli.”

It would be good to state if the clinical picture (low haemoglobin, low platelets, schistocytes) was present or not in any of the subjects.

2. Dialysis was unavailable and this contributed to the high mortality rates.

Under recommendations, would be good to add a note that Africa needs heightened advocacy and provision of organ support services in general and paediatric dialysis facilities in particular.
