# Supplementary material for: Causes and risk factors for an acute kidney injury outbreak among children in The Gambia, June – September 2022: A case-cohort study
Source: PLoS One. 2025 Jun 12;20(6):e0324931. doi: 10.1371/journal.pone.0324931 (PMC12161566; doi:10.1371/journal.pone.0324931)
Supplement: Attachment — Submitted filename: 25.10.24_RESPONSES TO REVIEWERS COMMENTS FINAL RESPONSE.docx [file pone.0324931.s006.docx]

**RESPONSES TO THE REVIEWERS**

PONE-D-24-22500R1

Causes and risk factors for an acute kidney injury outbreak among children in The Gambia, June – September 2022: A case-cohort study

PLOS ONE

Dear Dr. Office,

Thank you for submitting your manuscript to PLOS ONE. After careful consideration, we feel that it has merit but does not fully meet PLOS ONE’s publication criteria as it currently stands. Therefore, we invite you to submit a revised version of the manuscript that addresses the points raised during the review process.

Please submit your revised manuscript by Oct 31 2024 11:59PM. If you will need more time than this to complete your revisions, please reply to this message or contact the journal office at [plosone@plos.org](mailto:plosone@plos.org). When you're ready to submit your revision, log on to <https://www.editorialmanager.com/pone/> and select the 'Submissions Needing Revision' folder to locate your manuscript file.

Please include the following items when submitting your revised manuscript:

- A rebuttal letter that responds to each point raised by the academic editor and reviewer(s). You should upload this letter as a separate file labeled 'Response to Reviewers'.
- A marked-up copy of your manuscript that highlights changes made to the original version. You should upload this as a separate file labeled 'Revised Manuscript with Track Changes'.
- An unmarked version of your revised paper without tracked changes. You should upload this as a separate file labeled 'Manuscript'.

If you would like to make changes to your financial disclosure, please include your updated statement in your cover letter. Guidelines for resubmitting your figure files are available below the reviewer comments at the end of this letter.

If applicable, we recommend that you deposit your laboratory protocols in protocols.io to enhance the reproducibility of your results. Protocols.io assigns your protocol its own identifier (DOI) so that it can be cited independently in the future. For instructions see: [https://journals.plos.org/plosone/s/submission-guidelines#loc-laboratory-protocols](https://c05y1x9s.r.us-east-2.awstrack.me/L0/https:%2F%2Fjournals.plos.org%2Fplosone%2Fs%2Fsubmission-guidelines%23loc-laboratory-protocols/1/010f0191fcef1600-2c72adea-d022-46f8-939e-09afde656a06-000000/gLioirePI5OuDV2MvuPhfBHwPWE=176). Additionally, PLOS ONE offers an option for publishing peer-reviewed Lab Protocol articles, which describe protocols hosted on protocols.io. Read more information on sharing protocols at [https://plos.org/protocols?utm_medium=editorial-email&utm_source=authorletters&utm_campaign=protocols](https://c05y1x9s.r.us-east-2.awstrack.me/L0/https:%2F%2Fplos.org%2Fprotocols%3Futm_medium=editorial-email%26utm_source=authorletters%26utm_campaign=protocols/1/010f0191fcef1600-2c72adea-d022-46f8-939e-09afde656a06-000000/vGxoBzDAdoaUPckB0LXQhptuyDI=176).

We look forward to receiving your revised manuscript.

Kind regards,

Tungki Pratama Umar, M.D.
Academic Editor
PLOS ONE

**Editor Comments**:

Thank you for your effort to revise this manuscript following four reviewers and my comments. From the evaluation, most of the comments have been resolved, however, there are several concerns remained in this manuscript, both from the reviewers and I, which made a revise decision to be selected. Here are my comments, please also check all reviewers' comments.

- In the objective, add "in Gambia" since the risk factors is limited in this country
- After we collected primary data, add by what method (e.g. interview)
- Software used to generate Gambia map figure has not been written, also I think the authors deleted the caption (as shown by track changes). Or the authors plan to remove this figure?


[Note: HTML markup is below. Please do not edit.]

Reviewers' comments:

Reviewer's Responses to Questions

**Comments to the Author**

1. If the authors have adequately addressed your comments raised in a previous round of review and you feel that this manuscript is now acceptable for publication, you may indicate that here to bypass the “Comments to the Author” section, enter your conflict of interest statement in the “Confidential to Editor” section, and submit your "Accept" recommendation.

Reviewer #1: (No Response)

Reviewer #2: All comments have been addressed

Reviewer #3: All comments have been addressed

Reviewer #4: (No Response)

2. Is the manuscript technically sound, and do the data support the conclusions?

The manuscript must describe a technically sound piece of scientific research with data that supports the conclusions. Experiments must have been conducted rigorously, with appropriate controls, replication, and sample sizes. The conclusions must be drawn appropriately based on the data presented.

Reviewer #1: Partly

Reviewer #2: Yes

Reviewer #3: Yes

Reviewer #4: Yes

3. Has the statistical analysis been performed appropriately and rigorously?

Reviewer #1: No

Reviewer #2: I Don't Know

Reviewer #3: Yes

Reviewer #4: Yes

4. Have the authors made all data underlying the findings in their manuscript fully available?

The [PLOS Data policy](http://c05y1x9s.r.us-east-2.awstrack.me/L0/http:%2F%2Fwww.plosone.org%2Fstatic%2Fpolicies.action%23sharing/1/010f0191fcef1600-2c72adea-d022-46f8-939e-09afde656a06-000000/q2A5egOO9i_25UhCeVXvdwkFC4c=176) requires authors to make all data underlying the findings described in their manuscript fully available without restriction, with rare exception (please refer to the Data Availability Statement in the manuscript PDF file). The data should be provided as part of the manuscript or its supporting information, or deposited to a public repository. For example, in addition to summary statistics, the data points behind means, medians and variance measures should be available. If there are restrictions on publicly sharing data—e.g. participant privacy or use of data from a third party—those must be specified.

Reviewer #1: Yes

Reviewer #2: Yes

Reviewer #3: Yes

Reviewer #4: No

5. Is the manuscript presented in an intelligible fashion and written in standard English?

PLOS ONE does not copyedit accepted manuscripts, so the language in submitted articles must be clear, correct, and unambiguous. Any typographical or grammatical errors should be corrected at revision, so please note any specific errors here.

Reviewer #1: Yes

Reviewer #2: Yes

Reviewer #3: Yes

Reviewer #4: Yes

|  | **Comments** | **Response** | **Pages, lines** |
| --- | --- | --- | --- |
| 1 | Reviewer #1: Thanks for the rebuttal. I have following comments:  Also, authors should provide the p-value for baseline sociodemographic characteristics to show whether any of the baseline characteristics were different between the two groups.    Please mention what was the criteria for selecting factors for logistic regression analysis.  “In the identification of risk factors, by design, we do not look for the effect of an exposure on an outcome. Hence, there is nothing like adjusting for confounders.” The rebuttal is not right. The cause-and-effect relationship will be meaningful only if one adjusts for the baseline confounders which are very well present in the study. The groups developing AKI also had higher use of anti-inflammatory drugs, antibiotics, acetaminophen. How do we know that these were not the causes of the AKI rather than the suspected adulterated cough syrups. To say that the adulterated drug was the culprit authors should adjust for these factors in the study. Authors are not looking for just risk factors. They are putting a causal association with adulterated drug and hence it becomes important. “The acute kidney injury outbreak that occurred among children in The Gambia in the period June through September 2022 was causally associated with the ingestion of adulterated medicines.” | Thank you for your comment. The practice of providing p-values for comparing baseline sociodemographic characteristics to determine differences between groups is considered outdated, as evidenced by the references below. Use of p-values to compare baseline characteristics is not appropriate because:   1. There is no hypothesis to test regarding these baseline characteristics. 2. Inferential measures such as standard errors and confidence intervals should not be used to describe the variability of characteristics, and significance tests should be avoided in descriptive tables.   (Vandenbroucke, Jan P. et al., 2007. For the STROBE Initiative. Strengthening the Reporting of Observational Studies in Epidemiology (STROBE): Explanation and Elaboration. Epidemiology 18(6):p 805-835, November 2007. \| DOI: 10.1097/EDE.0b013e3181577511  Dales LG, Ury HK. An improper use of statistical significance testing in studying covariables. *International Journal of Epidemiology*. 1978;7:373–5)  Thank you for your comment. We used logistic regression for the **second objective** to identify risk factors of the AKI outbreak.  We used the backward logistic regression model to identify risk factors for the AKI outbreak. This procedure is good for exploratory data analysis to identify the most influential variables when there is little prior knowledge about the data. This was the case for this outbreak. Backward logistic regression helps to systematically remove variables that do not significantly contribute to the model, which includes confounders.  We used a conservative cut-off p-value of ≤ 0.20 for elimination of non-influential variables and used a cut-off p-value of ≤ 0.05 to determine the final risk factors.  By doing so, the procedure retained only the most relevant predictors thereby reducing the potential bias introduced by confounders.  Important to note that for this objective, we were not assessing for a causal association but just identifying factors that are associated with the outcome. The causal association of ingestion of adulterated medicines (main exposure) with AKI was determined using the MSM which by design controls for confounding. | No change in the text  No change in the text |
|  |  |  |  |
|  | Reviewer #4: The manuscript reads very well. Methods: 1. For the definition of controls: Were these all well children from the community from which the AKI cases originated or were some of them unwell with some other sickness at the time of the outbreak? Kindly clarify this in the definition of controls.  2. Line 129-130: “fever, vomiting, 130 diarrhea, cough, with history of syrup consumption or a child 8 years old”  This was used as one of the three definitions of a suspected AKI case.  This is too imprecise a definition for AKI since no renal specific symptoms are included in this definition. The mere fact that a child consumed syrup would be too imprecise to label them as suspected cases.  How many subjects were included because of this particular definition? If subjects recruited on the basis of this definition did not have AKI, their inclusion could skew the results.  [ Additionally from table 1/2 some of the controls also had vomiting, diarrhoea, cough and had ingested adulterated medications suggesting that the presence of renal specific symptoms in the definition is important] | The main criterion for being a control was that the child did not have AKI. However, we went ahead to assess for the presence of other illnesses that the controls had at the time when the event happened. This assessment was done for both cases and controls. So, our definition for the control remains the same.  Thank you for your observation. We agree that the first definition is very imprecise. This was the first definition and was later refined to the latter two. Most of the cases (49; 78%) were assessed using the criteria with renal specific symptoms (anuria/oliguria). Only 14 cases were assessed using the simple definition. However, they were confirmed by a nephrologist to have had AKI using other clinical criteria. | No change in the text  No change in the text |
|  | Reviewer #4:  Discussion:  1. Line 272-274 “The alternative explanation of E. coli infection is unlikely because only 8% of cases presented with bloody 273 diarrhea which increases the risk of developing serious complications such as acute renal failure. Only two 274 samples tested positive for Shiga toxin-producing E. coli.” It would be good to state if the clinical picture (low haemoglobin, low platelets, schistocytes) was present or not in any of the subjects if this data is available.  2. Dialysis was unavailable and this contributed to the high mortality rates. Under recommendations, would be good to add a note that Africa needs heightened advocacy and provision of organ support services in general and paediatric dialysis facilities in particular. | We agree with you on the need to add more clinical and laboratory characteristics. Unfortunately, these data were not collected and therefore not available since the study was done retrospectively.  Thank you for the suggestion. This has been incorporated. | No change in the text  Page 20, lines 368 - 369 |
