# Supplementary material for: Causes and risk factors for an acute kidney injury outbreak among children in The Gambia, June – September 2022: A case-cohort study
Source: PLoS One. 2025 Jun 12;20(6):e0324931. doi: 10.1371/journal.pone.0324931 (PMC12161566; doi:10.1371/journal.pone.0324931)
Supplement: Attachment — Submitted filename: 06.12.24_RESPONSES TO REVIEWERS COMMENTS 3rd ROUND.docx [file pone.0324931.s007.docx]

**RESPONSES TO THE REVIEWERS**

PONE-D-24-22500R2

Causes and risk factors for an acute kidney injury outbreak among children in The Gambia, June – September 2022: A case-cohort study.

PLOS ONE

|  | **Comments** | **Response** | **Pages, lines** |
| --- | --- | --- | --- |
|  | **Additional Editor Comments:**  Thank you for your effort to revise this manuscript following three reviewers' comments. However, I noted that the response to the editor comments are inadequate (not listed). Thus, please recheck my comment as also mentioned earlier.  - After we collected primary data, add by what method (e.g. interview) | Thank you for your comments. We collected primary data using interviewer-administered questionnaires and reviewed data from the national epidemiological surveillance system, line-list and preliminary epidemiological reports. | Line 109-110 |
|  | - Software used to generate Gambia map figures has not been written, also I think the authors deleted the caption (as shown by track changes). Or the authors plan to remove this figure? | Thank you so very much for your remarks. In our previous versions, the figure was deleted by mistake. We addressed it by bringing it back | Lines 116 – 117, Page 6 |
|  | **6. Review Comments to the Author**  Reviewer #3: The manuscript is very interesting. I have the following comment: references 4 and 8 are the same. Please review the numbering of the corrected references in the article. | Thank you so very much for the remark. We removed the double reference, checked for the accuracy of the references, and renumbered them appropriately | Lines 71 to 443; Pages 4 to 22 |
|  | Reviewer #4: "Only 14 cases were assessed using the simple definition. However, they were confirmed by a nephrologist to have had AKI using other clinical criteria."  Kindly provide a note in the manuscript stating that the first definition was later amended. | Thank you for your comment. We provided a sentence in the manuscript to address the comment. “The definition of a suspected case was revised to enhance its sensitivity by incorporating symptoms specific to renal conditions.” | Lines 135 to 136; |
